# Multiplex Immunoassay for Biomarker Profiling of Whole Blood Cell Lysates and Supernatants and Pathogen Response in Neat Whole Blood Cultures

**DOI:** 10.3390/mps8030046

**Published:** 2025-05-01

**Authors:** Irina Balan, Alejandro G. Lopez, A. Leslie Morrow

**Affiliations:** 1 Bowles Center for Alcohol Studies, School of Medicine, The University of North Carolina at Chapel Hill, Chapel Hill, NC 27599, USA; alopezsf@live.unc.edu; 2 Department of Psychiatry, School of Medicine, The University of North Carolina at Chapel Hill, Chapel Hill, NC 27599, USA; 3 Department of Pharmacology, School of Medicine, The University of North Carolina at Chapel Hill, Chapel Hill, NC 27599, USA

**Keywords:** biomarker profiling, cytokines, human whole blood cell culture, lipopolysaccharide, inflammatory pathways

## Abstract

Replicating in vivo conditions is essential for understanding immune responses and measuring immune biomarkers in blood. Sampling immune biomarkers in plasma or serum often fails to detect disease-relevant signals, possibly because these markers are sequestered in immune cells or extracellular vesicles. Furthermore, traditional whole blood cultures using external media may not accurately mimic the physiological environment of blood cells. To address these limitations, we developed a strategy using whole blood cell lysates and supernatants to optimize biomarker detection. Additionally, we employed neat whole blood culture methods, preserving the natural cellular and biochemical environment to assess sensitivity to immune modulators, such as lipopolysaccharide (LPS). This cost-effective approach minimizes variability and contamination risks. By utilizing Luminex multiplex immunoassays, we profiled immune biomarkers with higher sensitivity and efficiency than traditional ELISAs. Blood samples from individuals with high alcohol consumption validated our method by assessing biomarker levels before and after LPS stimulation, providing insights into intracellular responses and inflammatory pathways. This method enhances our understanding of inflammatory processes in blood cells, demonstrating the advantages of cell lysates, supernatants, and advanced multiplex assays in immunological research.

## 1. Introduction

In immunological research, the methods used to measure immune biomarkers in blood significantly impact the relevance and applicability of the findings. In this study, we introduce a refined approach for obtaining blood cell lysates and supernatants from whole blood before and after in vitro stimulation. This method provides deeper insights into intracellular mechanisms and cellular responses to external challenges under near-physiological conditions. The use of multiplex assays offers several advantages over traditional enzyme-linked immunosorbent assays (ELISAs), including the ability to simultaneously measure multiple biomarkers in a single sample, improving efficiency and minimizing sample consumption. This is particularly beneficial when working with limited blood volumes. Additionally, multiplex assays provide higher sensitivity and reduce variability between the assays, leading to more consistent and reliable results [1].

Traditionally, whole blood cultures are supplemented with external media to support cellular activity and viability over extended periods [2,3,4]. However, this approach may not accurately replicate the physiological conditions in which blood cells function in vivo. To address this limitation, neat whole blood culture methods, where blood is cultured without exogenous media, have been developed [5,6]. This approach preserves the natural biochemical and cellular environment of blood, maintaining conditions that closely mimic those found in the body. By avoiding external nutrients or growth factors, this method preserves the complex interplay between blood cells and plasma constituents, allowing for a direct assessment of cellular responses to stimuli or toxins. It is also simpler, more cost-effective, and reduces experimental variability and contamination risks.

The analysis of cytokine levels in clinical blood specimens is crucial for disease diagnosis and immune function monitoring. Cytokines serve as key signaling molecules in the immune system, and their fluctuations can indicate inflammation, infection, autoimmune disorders, or other pathological conditions [7]. Ensuring the stability of cytokines during sample collection and processing is essential for obtaining accurate data in both research and clinical diagnostics. Cytokines are highly sensitive to enzymatic degradation, temperature shifts, and handling conditions [8]. Minimizing handling time and maintaining samples at low temperatures slows enzymatic activity and preserves cytokine stability. Additionally, protease and phosphatase inhibitors are added to prevent enzymatic degradation. Protease inhibitors block protein breakdown, while phosphatase inhibitors preserve the phosphorylation states of signaling molecules, ensuring that cytokines and intracellular proteins retain their bioactivity and functional signaling capacity. Without these precautions, degradation may lead to an underestimation of cytokine levels, resulting in inaccurate measurements and potential misinterpretation of immune signals or responses.

Cytokines circulate in the bloodstream both as free molecules and encapsulated within extracellular vesicles (EVs), which protect them from enzymatic degradation [9]. To achieve a comprehensive cytokine profile, it is essential to lyse these EVs, releasing their cytokine content for accurate quantification in multiplex immunoassays. Lysis buffers containing detergents, such as a radioimmunoprecipitation assay (RIPA) buffer, which is commonly used to disrupt cellular and vesicular membranes, effectively releasing encapsulated cytokines from EVs. Studies have demonstrated that detergent-treated EVs in plasma leads to the release of their luminal cytokine content, facilitating accurate measurement in downstream analyses [10].

Sonication, which employs high-frequency sound waves, is another technique utilized to disrupt EV membranes. When applied under controlled conditions, sonication effectively lyses EVs without causing excessive heat generation, ensuring that both free and vesicle-bound cytokines become accessible for detection. This method enhances assay sensitivity and reliability, providing a more complete assessment of cytokine levels, which is critical for understanding immune and inflammatory dynamics [9].

To assess the robustness of this method, we refined our protocol using blood samples from individuals with high alcohol consumption, who are known to exhibit elevated levels of inflammatory biomarkers [11,12,13,14,15,16]. This model allows us to validate the performance of our blood cell lysate and supernatant method in conjunction with multiplex assays. Measuring biomarkers at baseline, post-culture, and after pathogen/toxin exposure provides insights into intracellular and extracellular biomarker levels, offering a clearer understanding of cellular responses to immune challenges, such as lipopolysaccharide (LPS). Additionally, cell lysates allow for the assessment of inflammatory receptor activation levels, including toll-like receptors, which play a central role in triggering inflammatory signaling pathways in individuals with alcohol use disorders (AUD) [17,18,19].

This comprehensive approach advances our understanding of how immune cells respond to pathogen or toxin exposure by providing detailed biomarker profiles at the cellular level. This method contributes valuable insights into immunology and toxicology while improving our ability to study disease processes and treatment effects in inflammatory and immune-related conditions.

## 2. Experimental Design

We previously used whole blood culturing, along with cell collection and lysis procedures, to measure inflammatory cytokine levels using ELISAs. These measurements were conducted in whole blood cell lysates before and after culturing for 4 h with LPS or imiquimod in postpartum depression patient samples, both before and after IV brexanolone administration [5]. This approach was effective in identifying immune biomarkers, such as IL-6 and TNF-α, that were inhibited by brexanolone and predicted HAM-D score improvement. However, the sensitivity of the ELISAs was limited, particularly for biomarkers like IL-1β at baseline levels. Using the ELISA for IL-1β (RayBiotech, Peachtree Corners, GA, USA; Cat. No.: ELH-IL1b), IL-1β was undetectable in the blood cell lysates of more than two-thirds of the participants. Additionally, due to the small volume of blood cell lysate available per condition, only four biomarkers could be measured using the ELISAs.

To address these limitations, we introduced methods to improve sample integrity and optimized immune marker detection in blood cell lysates and supernatants by employing Luminex multiplex immunoassays. Several key modifications were made to improve sample integrity. The time that blood was kept on ice during transfer from the hospital to the laboratory was reduced from one hour to less than 15 min to minimize blood cell degeneration. The lysis buffer was supplemented with protease and phosphatase inhibitors to prevent protein breakdown by proteolytic enzymes and to maintain the phosphorylation state of signaling proteins. Additionally, an advanced ultrasonic sonicator (Qsonica, Newtown, CT, USA; Part no.: Q500) with a cup horn (Qsonica, Newtown, CT, USA; Model no.: 431C2) was used for the sonication of blood samples. This system processes samples in sealed tubes to prevent aerosols and cross-contamination, allows for the simultaneous processing of multiple tubes, and maintains a cold temperature by adding ice to the cup horn, helping to prevent cytokine degradation, and ensuring higher total protein levels. Furthermore, sonication aids in the disruption of protein aggregates, ensuring a more uniform protein suspension and improving cytokine recovery by reducing the risk of insoluble protein complexes interfering with downstream analyses [8,20,21].

Luminex multiplex immunoassays utilize bead-based technology to enable the simultaneous detection of multiple biomarkers within a single sample, offering a high-throughput and sensitive approach for biomarker profiling. The assay uses uniquely color-coded microspheres, each conjugated with a specific captured antibody that binds to a target analyte. These beads are incubated with the biological sample, allowing antigen–antibody interactions to occur. A biotinylated detection antibody is then introduced, followed by streptavidin–phycoerythrin (PE), which serves as a fluorescent reporter for signal quantification. Using a dual-laser flow cytometry-based system, the Luminex instrument classifies each bead based on its internal fluorescence signature while simultaneously measuring the PE fluorescence intensity, which corresponds to the concentration of the bound analyte. This method allows for precise quantification of multiple biomarkers using a minimal sample volume, making it particularly useful for analyzing inflammatory and neuroinflammatory cytokines in blood cell lysates and supernatants [22]. This method significantly enhances sensitivity, allowing for the detection of biomarkers at low concentrations [1].

To maintain assay accuracy, it is important to minimize photobleaching, as prolonged exposure to light can degrade fluorescence signals and affect data integrity. Beads should be stored in opaque containers at 2–8 °C and protected from direct light to maintain fluorescence stability. Proper handling and storage conditions are necessary for ensuring the reproducibility and reliability of biomarker measurements using the Luminex platform.

Whole blood (~12 mL) was drawn from six participants with high alcohol consumption (four males and two females, with an average age and standard deviation (±SD) of 24.8 ± 4.0 years, ranging from 21 to 31 years old) and collected into sodium heparin-coated Vacutainer^®^ Plastic tubes (BD, Franklin Lakes, NJ, USA; Catalog No.: 367878). This study adhered to the Declaration of Helsinki and was approved by the Institutional Review Board of the University of North Carolina (UNC), Chapel Hill, NC, USA (protocol code 22-1164, date of approval: 26 May 2022). Informed consent was obtained from all participants. All participants were non-smokers and had no inflammatory-related diseases. Each participant was evaluated using the Alcohol Use Disorders Identification Test (AUDIT) and the Alcohol Use Disorders Identification Test—Consumption (AUDIT-C) prior to blood collection [23]. The mean AUDIT (±SD) was 13.3 (±6.0), and the mean AUDIT-C (±SD) was 7.5 (±1.4). Participants maintained their usual levels of physical activity, avoided any unusual or intense activities prior to blood collection, and adhered to their regular dietary habits. Blood samples were collected in the morning after an overnight fast to minimize circadian rhythm effects [8].

Immediately after blood collection, the heparin-coated tubes were gently inverted 8–10 times to prevent red blood cell breakage and ensure proper mixing with sodium heparin to prevent clotting. In this study, blood was placed on ice to minimize cell degeneration and kept on ice for less than 15 min during transfer to the laboratory. However, in our previous work involving cell lysate samples, blood was kept on ice for approximately one hour during transport without compromising cytokine detection [5]. Given that biomarker levels are lower in lysates compared to supernatants, the protocol remains adaptable to longer transfer times when earlier processing is not possible.

To measure baseline marker levels, 1 mL of blood was added to each of three 2 mL tubes, followed by 1 mL of a RIPA lysis buffer supplemented with protease and phosphatase inhibitors, along with an additional 10 µL of each type of inhibitor. Samples were centrifuged to obtain cell pellets, supernatants were collected, frozen on dry ice, and stored at −80 °C. Cell pellets were re-suspended in 150 µL of the RIPA buffer with protease and phosphatase inhibitors, sonicated, centrifuged, frozen on dry ice, and stored at −80 °C.

A RIPA buffer is widely used for effective cell lysis and extraction of intracellular proteins, including cytokines, in various immunoassays. It enhances cytokine detection by releasing intracellular, membrane-associated, and extracellular vesicle-encapsulated cytokines, thereby increasing the levels of detectable free cytokines. This is particularly beneficial when analyzing cell lysates, as the RIPA buffer efficiently solubilizes cellular and vesicular components, ensuring higher cytokine recovery and improved sensitivity in downstream analyses [10,24].

The RIPA buffer used in this protocol (Catalog Number: R0278, Sigma-Aldrich, St. Louis, MO, USA) contains 150 mM NaCl, 1.0% IGEPAL^®^ CA-630, 0.5% sodium deoxycholate, 0.1% SDS, and 50 mM Tris (pH 8.0). This combination of nonionic (IGEPAL), anionic (SDS, deoxycholate), and ionic (NaCl) compounds efficiently lyses leukocytes and EVs while solubilizing membrane-bound cytokines, enhancing their detection in Luminex assays. The IGEPAL^®^ CA-630 component acts as a mild nonionic detergent, facilitating cell membrane solubilization while minimizing protein denaturation. Sodium deoxycholate and SDS contribute to cell lysis and membrane disruption, promoting the release of cytokines from intracellular compartments and EVs, thereby increasing their availability for detection.

However, while the RIPA buffer effectively releases cytokines, its anionic detergents, SDS, and sodium deoxycholate, may affect bead-based immunoassays, potentially influencing antibody interactions and overall assay performance, leading to some inconsistencies in signal detection. Although IGEPAL^®^ CA-630 is a mild nonionic detergent and does not typically impact antibody interactions, the presence of SDS and deoxycholate requires attention during sample handling and processing to minimize potential interference. To address these effects while preserving the cytokine-releasing benefits of the RIPA buffer, dilution and preincubation are important for ensuring compatibility with the Luminex assays.

An important step in our Luminex immunoassay protocol is the use of a Platinum buffer (Catalog Number: EPXP-11113-000, Thermo Fisher Scientific, Vienna, Austria) instead of the standard assay buffer provided in the Luminex kit. While the Luminex assay buffer is designed for plasma and serum samples, it is not optimized for processing cell lysates that contain detergents like the RIPA buffer. The Platinum buffer is designed to reduce detergent interference while supporting cytokine solubility and antibody binding efficiency. It provides better compatibility with the RIPA-treated lysates, ensuring that residual detergents do not interfere with the bead-based assay. Additionally, preincubation of the RIPA-treated lysates with the Platinum buffer stabilizes cytokine–antibody interactions, improving assay sensitivity and reducing signal variability.

For experiments with LPS stimuli, 1 mL of blood was added to each of six wells of low-adhesion 24-well plates. In each well, 5 µL of cell culture-specific protease inhibitors (Sigma-Aldrich, St. Louis, MO, USA, Cat. no.: P1860) were added. In the three wells, LPS (10 µg/mL) was added, and the remaining three wells served as unstimulated in vitro controls. Blood was cultured for 4 h in a cell culture incubator (37 °C, 5% CO_2_). The LPS concentration was previously validated to produce pronounced effects without causing blood cell death. The 4-h time point was selected based on prior findings showing significant cytokine responses without leading to cell death [5]. Cell collection and lysate preparation followed the same protocol as for baseline biomarker measurement.

Biomarker measurements were performed using multiplex immunoassays (ProcartaPlex 14-plex Kit, Thermo Fisher Scientific, Vienna, Austria) and the Luminex 200 instrument system (Thermo Fisher Scientific, Carlsbad, CA, USA). For the assays, 50 µL of standards, cell lysates, and supernatants were loaded per well of a 96-well plate. Biomarker levels were determined using standard curves generated from 4-fold serial dilutions of standards and analyzed with a 5-parameter logistic (5-PL) regression model in the Thermo Fisher ProcartaPlex Analysis App. Since the same volumes of samples (lysates and supernatants) and standards were used, and both samples and standards were diluted identically during the assay, no additional dilution correction was required during interpolation.

To express biomarker levels relative to the original volume of whole blood, the following correction factors were applied: (1) for cell lysates, the cell pellet from 1 mL of whole blood was resuspended in 150 µL of the RIPA buffer; thus, 50 µL (the volume used in the assay) corresponds to 333.3 µL of whole blood, and measured concentrations were multiplied by 0.15; (2) for supernatants, which underwent a 2-fold dilution with the RIPA buffer, measured concentrations were multiplied by 2 to adjust for the dilution factor. While multiplex immunoassays and refined protocols have led to significant improvements, variability can still be introduced through handling and processing of human blood samples. Further validation with larger sample sizes will help assess the reliability and robustness of these methods. Additionally, the use of sonication has improved protein solubilization by disrupting protein aggregates, ensuring a more uniform suspension and improving cytokine recovery in blood cell lysates.

### 2.1. Materials

1. Protease Inhibitor Cocktail for use in tissue culture (Sigma-Aldrich, St. Louis, MO, USA, Catalog Number: P1860)
2. Protease Inhibitor Cocktails (Sigma-Aldrich, St. Louis, MO, USA, Catalog Number: P8340)
3. Phosphatase Inhibitor Cocktail 3 (Sigma-Aldrich, St. Louis, MO, USA, Catalog Number: P0044)
4. Lipopolysaccharide suitable for cell culture (Sigma-Aldrich, St. Louis, MO, USA, Catalog Number: L7770)
5. Radioimmunoprecipitation (RIPA) buffer (Sigma-Aldrich, St. Louis, MO, USA, Catalog Number: R0278)
6. ProcartaPlex 14-plex Kit: Catalog Number: PPX-14-MXCE7DR, Lot #: 433427-000, Expiry date: 2025-12 (Thermo Fisher Scientific, Vienna, Austria)
  - Standard Mix A: Lot 388185-000, Expiry date: 01/12/2025
  - Standard Mix B: Lot 420280-000, Expiry date: 01/12/2025
  - Standard Mix C: Lot 422159-000, Expiry date: 01/12/2025
  - Standard Mix E: Lot 397396-000, Expiry date: 01/12/2025
  - Standard Mix 10: Lot 381352-000, Expiry date: 01/12/2025
  - 14-plex Beads: Lot 432882-000 Expiry date: 01/12/2025
  - 14-plex Detection Antibody: Lot 432883-000 Expiry date: 2025-12
  - Wash Buffer (10×): Component # WBEX/28, Lot 24022582
  - Streptavidin–PE: Component # SA-PE, Lot 404758-000
  - Flat bottom 96-well Plate (Black): Component # SVM182
  - Black Microplate Lid: Component # SVM104
  - Plate Covers, Component #SVM16
7. Platinum Assay Buffer (1×): Catalog Number: EPXP-11113-000, Lot number: 423690-000 (Thermo Fisher Scientific, Vienna, Austria)
8. Hand-Held Magnetic Plate Washer: Catalog Number: EXP-55555-000, Lot#20190429 (Thermo Fisher Scientific)
9. Costar^®^ 24-well Clear Flat Bottom Ultra-Low Attachment Multiple Well Plates, Individually Wrapped, With Lid, Sterile (Corning, Glendale, AZ, USA, Catalog Number: 3473)
10. Sodium heparin-coated Vacutainer^®^ Plastic Tubes (BD, Franklin Lakes, NJ, USA; Catalog Number: 367878)
11. Falcon^®^ 50 mL High Clarity Polypropylene Centrifuge Tube, Conical Bottom, Sterile (Corning, Reynosa, Tamaulipas, Mexico, Catalog Number: 352098)
12. Eppendorf™ Safe-Lock Tubes 2.0 mL—Microtube (Thermo Fisher Scientific, Waltham, MA, USA, Catalog Number: 05-402-7)
13. Tubes, 0.2 mL, flat cap (Thermo Fisher Scientific, Waltham, MA, USA, Catalog Number: AB0620)
14. Reversible Microtube Racks with Lid (Thermo Fisher Scientific, Waltham, MA, USA, Catalog Number: 8660)

### 2.2. Equipment

- Sonicator ultrasonic processor (Qsonica, Newtown, CT, USA; Part Number: Q500)
- Cup horn (Qsonica, Newtown, CT, USA; Model Number: 431C2)
- Thermo Scientific™ 1300 Series Class II, Type A2 Biological Safety Cabinet (Thermo Fisher Scientific, Carlsbad, CA, USA; Catalog Number: 1377)
- Luminex™ 200™ Instrument System (Thermo Fisher Scientific, Carlsbad, CA, USA; Catalog Number: APX10031)
- Forma™ Direct Heat CO_2_ Incubator (Thermo Fisher Scientific, Carlsbad, CA, USA; Catalog Number: 320)
- Eppendorf 5810 R refrigerated centrifuge (Eppendorf AG, Hamburg, Germany; Catalog Number: 5811000020)
- Sorvall™ Legend Micro 21R Centrifuge, Refrigerated (Thermo Fisher Scientific, Carlsbad, CA, USA; Catalog Number: 75002447)
- Digital Microplate Shaker (Thermo Fisher Scientific, Carlsbad, CA, USA; Catalog Number: 88882005)
- Basic Vortex Mixer (Thermo Fisher Scientific, Carlsbad, CA, USA; Catalog Number: 88882011)

## 3. Procedure

### 3.1. Baseline Blood Processing: Preparation of Blood Cell Lysates, Supernatants, and Plasma

#### 3.1.1. Blood Collection and Handling

- Collect 12 mL of whole blood per subject into sodium heparin-coated Vacutainer^®^ plastic tubes (BD, 367878).
- Immediately after collection, gently invert the tubes 8–10 times to ensure proper mixing with sodium heparin and prevent clotting.
- Place the tubes on ice immediately to minimize blood cell degeneration.
- Maintain blood on ice for less than 15 min while transferring it from the hospital to the laboratory for processing.

#### 3.1.2. Sample Preparation for Blood Cell Lysates and Supernatants

- Label six 2 mL tubes in advance as follows:
  - Control (CTL) 0 h Supernatants: CTL 0 h #1, CTL 0 h #2, CTL 0 h #3
  - Control (CTL) 0 h Cell Lysates: CTL 0 h #1, CTL 0 h #2, CTL 0 h #3
- Prepare the fresh cold RIPA buffer (~15 min in advance) and keep on ice:
  - 10 mL of the RIPA buffer (150 mM NaCl, 1.0% IGEPAL^®^ CA-630, 0.5% sodium deoxycholate, 0.1% SDS, 50 mM Tris, pH 8.0; Sigma-Aldrich, St. Louis, MO, USA, Catalog Number: R0278)
  - 100 µL of the protease inhibitor cocktail (1:100; Sigma-Aldrich, St. Louis, MO, USA, Catalog Number: P8340)
  - 100 µL of the phosphatase inhibitor cocktail 3 (1:100; Sigma-Aldrich, St. Louis, MO, USA, Catalog Number: P0044)

CRITICAL STEP: Prepare the fresh cold RIPA buffer before use (~15 min in advance) and keep it on ice throughout the experiment to prevent protein degradation. Use immediately to maintain enzymatic inhibitor activity, and to ensure optimal cell lysis efficiency.

c. Using sterile serological pipettes, on ice, gently add 1 mL of whole blood into each labeled ‘Cell Lysate’ tube.
  Add 10 µL of the protease inhibitor cocktail (1:100, Sigma-Aldrich, St. Louis, MO, USA, Catalog Number: P8340) and 10 µL of the phosphatase inhibitor cocktail 3 (1:100, Sigma-Aldrich, St. Louis, MO, USA, Catalog Number: P0044) to each tube.
d. Add 1 mL of the cold RIPA buffer with the protease and phosphatase inhibitors to each tube.
e. Vortex briefly for 2–3 s and centrifuge at 9500× *g*, 4 °C, for 5 min.
f. Carefully collect the supernatant (on ice) into the ‘Supernatant’ tubes, avoiding the pellet.
g. Place ‘Supernatant’ tubes on dry ice, then transfer them to a −80 °C freezer.
h. Remove remaining liquid from the ‘Cell Lysate’ tubes by gently tapping them onto absorbent paper to eliminate residual blood.
i. Return the tubes to the ice and add 150 µL of the cold RIPA buffer (with the protease and phosphatase inhibitors).
j. Vortex for 30 s and incubate on ice for 15 min.
k. Sonication: using an ultrasonic processor with a cup horn water bath (1/3 ice, 2/3 cold water), sonicate each tube twice for 30 s at 40% amplitude, ensuring a 30-s cooling period on ice between sonication steps to prevent overheating.
l. Centrifuge at 15,000× *g*, 4 °C, for 30 min.
m. Transfer the supernatant to new ‘Cell Lysate’ tubes, place them on dry ice, and store at −80 °C for further analysis.

#### 3.1.3. Plasma Preparation

- Centrifuge one sodium heparin-coated Vacutainer^®^ Plastic tube containing 2–3 mL of whole blood using an Eppendorf 5810 R centrifuge at 2000× *g*, 4 °C, for 15 min (acceleration 9, brake 0). Total centrifugation time: ~26 min.
- On ice, use a 1000 µL pipette with barrier tips to carefully collect plasma, avoiding the buffy coat.
- Aliquot 0.5 mL of plasma into labeled 1.5 mL tubes.
- Add 5 µL of the protease inhibitor cocktail (1:100, Sigma-Aldrich, St. Louis, MO, USA, Catalog Number: P8340) and 5 µL of the phosphatase inhibitor cocktail 3 (1:100, Sigma-Aldrich, St. Louis, MO, USA, Catalog Number: P0044) to each tube.
- Immediately place the tubes on dry ice, then transfer them to a −80 °C freezer.

### 3.2. Neat Whole Blood Culture and In Vitro Stimulation of Whole Blood by Lipopolysaccharide (LPS) and Sample Processing

#### 3.2.1. Neat Whole Blood Culture Preparation and LPS Stimulation

- Within 15 min after blood drawing, place a 24-well culture plate (Corning, Glendale, AZ, USA, Catalog Number: 3473, ultra-low attachment surface) on ice inside a biosafety cabinet to maintain sterility.
- Label six wells as follows:
  - Control (CTL) 4 h: CTL 4 h #1, CTL 4 h #2, CTL 4 h #3
  - LPS 4 h: LPS 4 h #1, LPS 4 h #2, LPS 4 h #3
- Using sterile serological pipettes, gently add 1 mL of whole blood into each well.
- Add 5 µL of the cell culture protease inhibitor (Sigma-Aldrich, St. Louis, MO, USA, Catalog Number: P1860, 1:200) to each well and gently mix by turning a pipette tip 1–2 circles inside the blood to distribute the inhibitor evenly.
- Add 10 µL of the LPS stock solution (1 mg/mL in sterile water) to the designated LPS wells to achieve a final concentration of 10 µg/mL. Again, gently mix by turning a pipette tip 1–2 circles inside the blood.

CRITICAL STEP: Keep the culture plate on ice under sterile conditions. The LPS stock solutions must be prepared and aliquoted under sterile conditions and stored at −20 °C. Do not shake the plate. Instead, gently mix the blood by rotating a pipette tip 1–2 times inside the well.

f. Gently transfer the plate to a cell culture incubator (37 °C, 5% CO_2_) and incubate for 4 h.

#### 3.2.2. Post-Incubation Sample Processing

- After 4 h, place the plate on ice and transfer it back to the biosafety cabinet for further processing.
- Label the following tubes in advance (3 of each type):
  - ‘CTL 4 h Supernatant’
  - ‘CTL 4 h Cell Lysate’
  - ‘LPS 4 h Supernatant’
  - ‘LPS 4 h Cell Lysate’
- Prepare the fresh cold RIPA buffer (~15 min in advance) and keep on ice:
  - 20 mL of the RIPA buffer (Sigma-Aldrich, St. Louis, MO, USA, Catalog Number: R0278)
  - 200 µL of the protease inhibitor cocktail (1:100, Sigma-Aldrich, St. Louis, MO, USA, Catalog Number: P8340)
  - 200 µL of the phosphatase inhibitor cocktail 3 (1:100, Sigma-Aldrich, St. Louis, MO, USA, Catalog Number: P0044)
- Add 10 µL of the protease inhibitor cocktail (1:100, Sigma-Aldrich, St. Louis, MO, USA, Catalog Number: P8340) and 10 µL of the phosphatase inhibitor cocktail 3 (1:100, Sigma-Aldrich, St. Louis, MO, USA, Catalog Number: P0044) to each well. Mix with a pipette tip.
- Add 1 mL of the cold RIPA buffer (with inhibitors) to each well containing 1ml of blood and mix using a pipette tip.
- Transfer the contents into the ‘Cell Lysate’ tubes (placed on ice).

The following steps are the same as in Section 3.1.2 (e–m).

#### 3.2.3. Biosafety Cleanup

- Disinfect the biosafety cabinet with 70% ethanol or an appropriate disinfectant.
- Dispose of biohazardous waste following institutional biosafety guidelines.

### 3.3. Luminex Assay Protocol for 14-Plex Beads

#### 3.3.1. Prepare Antigen Standards on Ice

- Centrifuge each antigen standard (Std) vial at 2000× *g* for 10 s.
- Add 50 µL of 1× the Platinum assay buffer to each antigen standard vial.
- Gently vortex for 10 s and centrifuge at 2000× *g* for 10 s to collect the contents.
- Incubate on ice for 10 min to ensure complete reconstitution.
- Pool the contents of all vials (total 5 vials) into one and fill with the Platinum assay buffer to a total volume of 250 µL.
- Gently vortex for 10 s and centrifuge at 2000× *g* for 10 s to collect the contents. This original antigen standard vial contains Std1.

##### Perform a 4-Fold Standard Serial Dilution

- Add 50 µL from the original antigen standard vial (Std1) to Std2 and add 150 µL of the Platinum assay buffer.
- Continue diluting for vials Std3–Std9 by transferring 50 µL from one vial to the next with 150 µL of the Platinum buffer in each.
- Mix each vial thoroughly by pipetting up and down 10 times.

#### 3.3.2. Prepare Dilutions for Blood Cell Lysate and Supernatant Samples

- For each condition, add 50 µL of cell lysates or supernatants into 0.2 mL tubes and add 100 µL of the Platinum buffer.
- Preincubate for 2 h at 4 °C while shaking at 600 rpm.

CRITICAL STEP: Cell lysates and supernatants prepared with the RIPA buffer are diluted 3-fold in the Platinum buffer before analysis. This dilution reduces detergent interference while maintaining high cytokine recovery. A preincubation step (2 h at 4 °C while shaking at 600 rpm) further optimizes cytokine–antibody interactions, improving assay sensitivity and reproducibility.

#### 3.3.3. Assay Protocol

- Plate Setup and Wash Buffer Preparation

- Define plate map: standards, background, and samples

b. Wash Buffer Preparation

- Bring Wash buffer (10×) to room temperature and vortex for 15 s.
- Add 5 mL of the Wash buffer concentrate (10×) to 45 mL of double-distilled water (ddH_2_O).
- Store at 2–8 °C for up to 6 months.

c. Bead Preparation

- Vortex the 1× Capture Bead Mix vial for 30 s at high speed.
- Add 50 µL of the Capture Bead mix to each well of the 96-well plate.

CRITICAL STEP: Beads should be stored in opaque containers at 2–8 °C and protected from direct light exposure, as fluorescent signals can diminish within 10 min under direct sunlight, 30 min to 1 h under incandescent light, and up to 4 h under fluorescent lighting. During handling and incubation, covering plates with black microplate lids and tubes with foil and using low-light conditions helps maintain bead fluorescence.

d. Wash Magnetic Beads

- Insert the 96-well plate with beads into the hand-held magnetic plate washer and allow beads to settle for 2 min.
- Remove liquid from the wells by inverting the magnetic plate over the waste container. Blot the plate on paper towels.
- Add 150 µL of the Wash buffer (1×) to each well, wait 2 min, and repeat the washing procedure.
- Remove the 96-well plate with beads from the magnetic plate washer.

e. Add Standards, Background, and Samples

- For standards and background: add 100 µL of the Platinum buffer and then 50 µL of the standards or background (Platinum buffer) to the appropriate wells of the 96-well plate.
- For blood cell lysates and supernatants: add 150 µL of the preincubated samples (see Section 3.3.2) to the respective wells of the 96-well plate.

f. Plate Sealing and Incubation

- Seal the plate with the provided plate seal and cover it with the black microplate lid.
- Incubate the plate while shaking at 600 rpm for 30 min at room temperature.
- Transfer the plate to 4 °C while shaking at 600 rpm overnight, ensuring it is protected from light.

g. Warm-Up and Wash the Plate

- In the morning, shake the plate at 600 rpm for 30 min to warm it to room temperature.
- Wash the 96-well plate twice using the magnetic plate washer (see Section 3.3.3 (d)).

h. Add Detection Antibody Mixture

- Add 25 µL of the Detection Antibody Mixture (1×) to each well.
- Seal the plate and cover it with the black microplate lid.
- Incubate for 1 h on a shaker at 600 rpm at room temperature.

i. Streptavidin–PE Addition

- Wash the plate twice using the magnetic plate washer (see Section 3.3.3 (d)).
- Add 50 µL of Streptavidin–PE to each well.
- Seal and incubate for 30 min on a shaker at 600 rpm at room temperature.

j. Final Plate Wash and Reading

- Wash the plate twice using the magnetic plate washer (see Section 3.3.3 (d)).
- Add 120 µL of the Wash buffer (1×) to each well for reading.
- Seal and incubate for 5 min on a shaker at 600 rpm at room temperature.
- Remove the plate seal and run the plate on a Luminex-200 instrument.

CRITICAL STEP: Use the Wash buffer (1×) instead of the Reading buffer to prevent bead aggregation, to minimize background noise, and to ensure accurate fluorescence detection. The Wash buffer maintains proper bead suspension and reduces interference, ensuring reliable assay performance.

PAUSE STEP: If the assay cannot be immediately run on the Luminex-200 instrument, preserve bead integrity and fluorescence signals by covering the plate with a lid or aluminum foil and storing it at 4 °C. For delays under 4 h, keep beads in suspension by gently mixing before resuming the assay. For overnight storage (12–24 h), seal the plate with an adhesive plate sealer, store at 4 °C in the dark, and allow it to reach room temperature for 30 min before measurement. Resuspend beads by shaking at 600 rpm for 5 min on a plate shaker. Do not freeze the assay, as this may damage the beads.

k. Data Analysis Using ProcartaPlex Luminex Thermo Fisher App

- Open the ProcartaPlex Luminex App and upload the raw xPONENT CSV files containing fluorescence intensity data.
- The app will automatically assign analytes to their corresponding bead regions.
- Verify and adjust the standard curves, ensuring proper curve fitting for accurate quantification.
- Apply dilution factors, sample layouts, and normalization as needed.
- Inspect the real-time plots of the standard curves, flag potential outliers, and confirm sample quantifications.
- Save the analyzed data in Excel, CSV, or PDF format for further statistical analysis and reporting.

## 4. Expected Results

Our method of measuring biomarkers in both cell lysates and supernatants provides a more comprehensive and sensitive assessment compared to traditional plasma-based analysis. Plasma alone may not fully reflect immune activity, as many cytokines and inflammatory mediators are stored inside immune cells or encapsulated in extracellular vesicles, rather than freely circulating. By analyzing both intracellular and extracellular compartments, we detect sequestered cytokines that would otherwise be missed, offering a more accurate representation of immune activation and signaling pathways.

The use of Luminex multiplex immunoassays in our study enabled the simultaneous quantification of multiple immune biomarkers with high sensitivity and minimal sample volume. This method offered a distinct advantage over traditional ELISAs, particularly in our whole blood-based approach where sample availability is limited. The overnight bead incubation further enhanced assay sensitivity, especially for low-abundance analytes, providing robust and reproducible cytokine measurements. The results confirm the utility of this platform in capturing the complex immune landscape in individuals with high alcohol consumption.

Plasma levels varied across biomarkers. Some, such as IL-1β, IL-3, IL-6, IL-17A, and TNF-α, were comparable to supernatant levels, while others, including BDNF, HMGB1, IL-7, IL-8, IL-18, and MCP-1, were several-fold lower in plasma. Additionally, IL-5, MIP-1β, and CCL11 were approximately 1.5 to 2 times lower in plasma than in supernatants (Table 1). These findings confirm that plasma-based biomarker analysis underestimates cytokine levels, particularly for intracellularly stored or vesicle-bound biomarkers, reinforcing the need to analyze both lysates and supernatants for a more complete immune profile.

LPS stimulation (10 µg/mL, 4 h) induced distinct biomarker responses in blood cell lysates and supernatants, demonstrating immune activation in a physiologically relevant whole blood environment. The most robust increases were observed in IL-1β, IL-6, IL-8, and MIP-1β, which were markedly elevated, particularly in supernatants, indicating strong immune activation and active cytokine release. The levels of TNF-α, IL-5, and IL-18 were also predominantly increased in supernatants, suggesting preferential secretion upon immune stimulation. By contrast, BDNF, HMGB1, IL-3, IL-7, IL-17A, MCP-1, and CCL11 remained unchanged, suggesting minimal responsiveness to LPS under these conditions (Table 2). These findings confirm that LPS primarily triggers the production and release of pro-inflammatory cytokines and chemokines, with higher levels in supernatants than in cell lysates. By employing neat whole blood culture instead of traditional diluted culture systems, we preserved the natural cellular and biochemical interactions of blood, providing a more accurate assessment of immune activation and inflammatory signaling pathways.

Efficient sample preparation was critical for maximizing cytokine detection. The addition of protease and phosphatase inhibitors prevented protein degradation and preserved the phosphorylation states of the signaling molecules. Sonication and the RIPA buffer synergistically improved cytokine recovery—sonication disrupted cellular and extracellular vesicle membranes, ensuring the release of sequestered cytokines and preventing protein aggregation, while the RIPA buffer lysed cells and solubilized membrane-bound proteins. Additionally, the Platinum buffer minimized assay interference, improving antibody binding and signal reproducibility in Luminex-based biomarker quantification. All steps were performed on ice to maintain protein integrity and to ensure accurate detection. In future studies with a larger and more diverse sample size, we aim to identify the immune biomarkers that may serve as indicators of AUD. By differentiating biomarker profiles between low and high alcohol drinkers, we will assess whether specific immune markers are associated with alcohol-related immune dysregulation and could help distinguish individuals with AUD. While the present study validated the protocol using samples from individuals with high alcohol consumption, future research should incorporate healthy control groups to further assess the sensitivity and dynamic range of the assay. Expanding the analysis to different populations will help confirm the broader applicability of the method across various physiological and pathological conditions.

Additionally, investigating sensitivity to LPS and other pathogen-associated stimuli will help clarify how blood cells respond to immune challenges across different drinking patterns, providing further insight into potential biomarkers of immune dysfunction in AUD. Future studies will benefit from incorporating additional immune stimulants beyond LPS, such as phytohemagglutinin or phorbol myristate acetate [25], to activate distinct signaling pathways and more comprehensively characterize immune responsiveness. Like LPS, IL-1 is a potent inducer of NF-κB signaling and plays a critical role in promoting inflammatory responses [26]. Including IL-1 as an alternative stimulant could further expand our understanding of immune activation dynamics in whole blood cultures.

We also recognize that soluble cytokine receptors present in plasma can bind free cytokines and potentially affect assay signals [27]. Although our protocol is designed to minimize this confounding factor by focusing on cellular and vesicle-associated cytokines, future studies could be further enhanced by quantifying soluble receptor levels or by incorporating complementary strategies to assess cytokine-receptor dynamics [28]. Beyond alcohol-related immune modulation, this protocol is highly relevant for clinical and translational research in inflammatory diseases. By assessing both intracellular and extracellular biomarker levels, our approach provides a comprehensive view of immune activation, inflammatory pathways, and regulatory mechanisms. Furthermore, the observed sensitivity of blood cells to pathogen-associated stimuli suggests that this method can be applied to various conditions, including autoimmune disorders, chronic infections, toxin-related immune dysregulation, neuropsychiatric disorders, neurodegenerative disorders, and immunotherapy research.

## Figures and Tables

**Table 1:** Comparative Analysis of 14-Plex Immunoassay Biomarker Levels in Blood Cell Lysates, Supernatants, and Plasma from Individuals with High Alcohol Consumption. This table presents the quantification of 14 biomarkers measured in picograms per milliliter (pg/mL) in blood cell lysates, supernatants, and plasma from six individuals categorized as high alcohol consumers. The results demonstrate differences in biomarker levels across the three compartments, with supernatants generally showing higher concentrations compared to cell lysates or plasma.

Biomarkers	Cell Lysates		Supernatants		Plasma	
	Mean	SEM	Mean	SEM	Mean	SEM
BDNF	75.12	20.07	1700.47	479.53	10.96	2.51
CCL11	3.99	0.97	116.33	27.83	50.91	4.91
HMGB1	1070.26	301.18	225,807.8	42,814.3	3558.36	1672.15
IL-1β	0.65	0.14	11.18	2.83	10.56	3.29
IL-3	0.72	0.34	68.81	48.7	60.25	15.91
IL-5	0.26	0.01	5.11	3.12	2.93	0.81
IL-6	0.65	0.14	3.05	1.04	2.46	0.91
IL-7	0.02	0.004	2.87	0.43	0.34	0.15
IL-8	1.1	0.41	5.9	1.57	0.30	0.16
IL-17A	0.13	0.03	4.98	2.24	5.84	2.34
IL-18	68.25	32.01	824.81	145.29	133.70	19.13
MCP-1	80.67	13.8	1298.58	296.7	314.72	65.25
MIP-1β	1.48	0.34	76.05	17.98	42.63	9.49
TNF-α	0.8	0.43	22.95	14.22	35.05	12.63

BDNF: brain-derived neurotrophic factor; CCL11: C-C motif chemokine ligand 11 (eotaxin-1); HMGB1: high mobility group box 1; IL-1β: interleukin 1 beta; IL-3: interleukin 3; IL-5: interleukin 5; IL-6: interleukin 6; IL-7: interleukin 7; IL-8: interleukin 8 (CXCL8); IL-17A: interleukin 17A; IL-18: interleukin 18; MCP-1: monocyte chemoattractant protein 1 (CCL2); MIP-1β: macrophage inflammatory protein 1 beta (CCL4); TNF-α: tumor necrosis factor alpha; SEM—standard error of the mean.

**Table 2:** Effects of Lipopolysaccharide (LPS) Treatment on Biomarker Levels in Blood Cell Lysates and Supernatants from Six Individuals with High Alcohol Consumption. This table presents the quantification of 14 biomarkers (pg/mL) measured in blood cell lysates and supernatants using a 14-plex immunoassay. Mean and SEM values are reported for baseline (untreated) and LPS-treated (10 µg/mL, 4 h) conditions. The data illustrate biomarker responses to in vitro LPS stimulation, highlighting changes in biomarker levels following immune activation.

Biomarkers	Cell Lysates				Supernatants			
	Baseline In Vitro		LPS-Induced In Vitro		Baseline In Vitro		LPS-Induced In Vitro	
	Mean	SEM	Mean	SEM	Mean	SEM	Mean	SEM
BDNF	48.82	9.73	48.86	8.00	1928.55	448.99	1739.00	441.25
CCL11	2.70	0.52	2.82	0.52	75.61	19.77	92.21	19.72
HMGB1	6055.51	538.94	6073.42	1466.23	323,592.92	33,967.29	279,486.67	30,428.08
IL-1β	14.91	2.97	90.16	14.40	874.83	139.40	5116.55	962.42
IL-3	1.01	0.85	0.33	0.13	41.68	30.14	48.08	34.79
IL-5	0.21	0.08	0.53	0.17	8.81	2.06	25.05	5.85
IL-6	13.12	5.12	221.99	52.97	2128.47	1098.80	16,590.56	3766.35
IL-7	0.03	0.01	0.10	0.02	1.96	0.33	2.77	0.41
IL-8	33.03	7.00	67.49	7.83	2699.08	412.33	5088.91	663.33
IL-17A	0.04	0.02	0.05	0.01	3.35	1.30	5.68	2.29
IL-18	6.88	1.24	7.60	1.66	535.49	132.12	2712.32	1869.04
MCP-1	52.42	10.54	37.59	9.20	4082.80	1162.86	2854.52	875.79
MIP-1β	26.72	8.00	80.26	9.30	1514.85	477.65	7088.30	2034.22
TNF-α	0.40	0.36	0.70	0.53	20.63	17.18	129.70	112.84

BDNF: brain-derived neurotrophic factor; CCL11: C-C motif chemokine ligand 11 (eotaxin-1); HMGB1: high mobility group box 1; IL-1β: interleukin 1 beta; IL-3: interleukin 3; IL-5: interleukin 5; IL-6: interleukin 6; IL-7: interleukin 7; IL-8: interleukin 8 (CXCL8); IL-17A: interleukin 17A; IL-18: interleukin 18; MCP-1: monocyte chemoattractant protein 1 (CCL2); MIP-1β: macrophage inflammatory protein 1 beta (CCL4); TNF-α: tumor necrosis factor alpha; SEM—standard error of the mean.

## Data Availability

All raw data for this study is available from the authors by reasonable request.
